# Oviductal Extracellular Vesicles Improve Post-Thaw Sperm Function in Red Wolves and Cheetahs

**DOI:** 10.3390/ijms21103733

**Published:** 2020-05-25

**Authors:** Marcia de Almeida Monteiro Melo Ferraz, Jennifer Beth Nagashima, Michael James Noonan, Adrienne E. Crosier, Nucharin Songsasen

**Affiliations:** 1 Smithsonian National Zoo and Conservation Biology Institute, 1500 Remount Road, Front Royal, VA 22630, USA; nagashimaj@si.edu (J.B.N.); michael.noonan@ubc.ca (M.J.N.); crosiera@si.edu (A.E.C.); songsasenn@si.edu (N.S.); 2 The Irving K. Barber School of Arts and Sciences, The University of British Columbia, Okanagan Campus, 1177 Research Road, Kelowna, BC V1V 1V7, Canada

**Keywords:** wildlife, gamete rescue, oviduct, cryopreservation, sperm

## Abstract

Artificial insemination (AI) is a valuable tool for ex situ wildlife conservation, allowing the re-infusion and dissemination of genetic material, even after death of the donor. However, the application of AI to species conservation is still limited, due mainly to the poor survival of cryopreserved sperm. Recent work demonstrated that oviductal extracellular vesicles (oEVs) improved cat sperm motility and reduced premature acrosomal exocytosis. Here, we build on these findings by describing the protein content of dog and cat oEVs and investigating whether the incubation of cryopreserved red wolf and cheetah sperm with oEVs during thawing improves sperm function. Both red wolf and cheetah sperm thawed with dog and cat oEVs, respectively, had more intact acrosomes than the non-EV controls. Moreover, red wolf sperm thawed in the presence of dog oEVs better maintained sperm motility over time (>15%) though such an improvement was not observed in cheetah sperm. Our work demonstrates that dog and cat oEVs carry proteins important for sperm function and improve post-thaw motility and/or acrosome integrity of red wolf and cheetah sperm in vitro. The findings show how oEVs can be a valuable tool for improving the success of AI with cryopreserved sperm in threatened species.

## 1. Introduction

The red wolf (*Canis rufus*) is a critically endangered American canid [1], with fewer than 25 wild individuals remaining in a reintroduced population in North Carolina, United States, and approximately 262 managed individuals in the Species Survival Plan population [2]. The cheetah (*Acinonyx jubatus*) is classified as ‘vulnerable’ by the International Union for Conservation of Nature (IUCN) [1], and occurs mainly in isolated populations in northern, southern and eastern Africa. The cheetah experienced two historic population bottlenecks and, as a result, the species has low heterozygosity, which compromises the species overall fitness and reproduction [3,4,5]. The worldwide captive cheetah population is composed of approximately 1877 animals and, because of poor reproductive success and a high incidence of cub mortality (approximately 20%), is not self-sustaining [6]. It is vital to the survival of these species that every valuable animal is represented in the next generation in order to maintain genetic variability in the population [7,8]. Therefore, the primary goal of captive breeding is to maintain a species’ genetic diversity by ensuring that each individual’s genetic material is represented within the population [7]. While natural breeding is the gold standard, assisted reproductive technologies (ARTs), especially artificial insemination (AI), have played a pivotal role in the management of many endangered species, including the black-footed ferret, giant panda and whooping crane [7,9,10,11,12,13,14]. AI can eliminate the need for transporting individuals to different locations for breeding and failed reproduction due to sexual incompatibility [7,13,14]. When used in combination with semen cryopreservation, AI also allows re-infusion and dissemination of valuable genes even after death of the sperm donor [10,12]. The value of AI with cryopreserved sperm in endangered species conservation has been demonstrated in the black-footed ferret, African elephant, southern white rhinoceros, gray wolf, giant panda, clouded leopard and cheetah [11,15,16,17,18]. Specifically, the use of AI in the black-footed ferret (including by sperm that had been cryopreserved for 20 years) in the captive breeding program has increased population gene diversity by 0.2% and reduced inbreeding by 5.8% when compared with natural mating alone [12].

Despite the potential utility of ARTs, the practical application of AI to carnivores, including wild canids and felids, is still limited. This is mainly due to poor post-freeze and post-thaw sperm survival. The functionality of a spermatozoon generally depends on its motility [19], acrosomal integrity [20], and its ability to undergo capacitation and acrosomal exocytosis [21]. Previous studies have examined the impact of several cryobiological factors, including type and concentration of cryoprotectants as well as cooling and warming rates on cryosurvival of canid and felid sperm [22,23,24,25,26]. Although the findings from such studies have laid an important foundation for the development of sperm cryopreservation protocols, the quality of cryopreserved gametes is still much lower than that of fresh ejaculates, and thus are not suitable for AI. The motility of cryopreserved red wolf sperm decreases by approximately 50% compared to fresh ejaculates [24,27], and the motility index of cheetah sperm is reduced by approximately 30% after freezing and thawing [25,28]. Moreover, the numbers of red wolf and cheetah sperm with intact acrosomes are reduced by approximately 40 and 45%, respectively, after freezing and thawing [24,25,27,28]. Thus, there is still a need to improve post-thaw survival of wild canid and felid sperm before AI can be reliably incorporated into breeding programs.

After mating, sperm travel into the uterus, enter the oviduct, and attach to the epithelium of the isthmus, a process which is necessary for extending sperm viability within the female reproductive tract [29,30]. It has been shown that the oviduct facilitates sperm function by the secretion of extracellular vesicles (oEVs) containing regulatory molecules (proteins, peptides, RNA species, lipids and DNA fragments) that contribute to cell–cell communication [31,32,33,34]. In this regard, recent work by our laboratory has shown that cat (*Felis catus*) oEVs are enriched in proteins related to energy metabolism, membrane modification, and reproductive function [31]. We have also demonstrated that incubating cat sperm with oEVs improves motility and fertilizing capacity of the gamete as well as prevents premature acrosomal exocytosis in vitro [31]. Although the impact of oEVs on sperm function has been investigated in the mouse and human [33,34], and oEVs have been demonstrated to support post-thaw bovine sperm motility under capacitating conditions [35], there have been no studies on the influence of different reproductive stage oEVs on post-thaw sperm function. Therefore, in the present study, we build on our previous findings by (1) characterizing the protein content of dog (*Canis familiaris*) and cat oEVs recovered at different stages of the reproductive cycle and (2) determine whether incubation of red wolf and cheetah sperm with oEVs obtained from their domestic counterparts during thawing could improve their motility and maintain acrosome integrity in vitro.

## 2. Results

### 2.1. Isolation and Characterization of Dog and Cat Oviductal Extracellular Vesicles (oEVs) 

**Figure 1:**
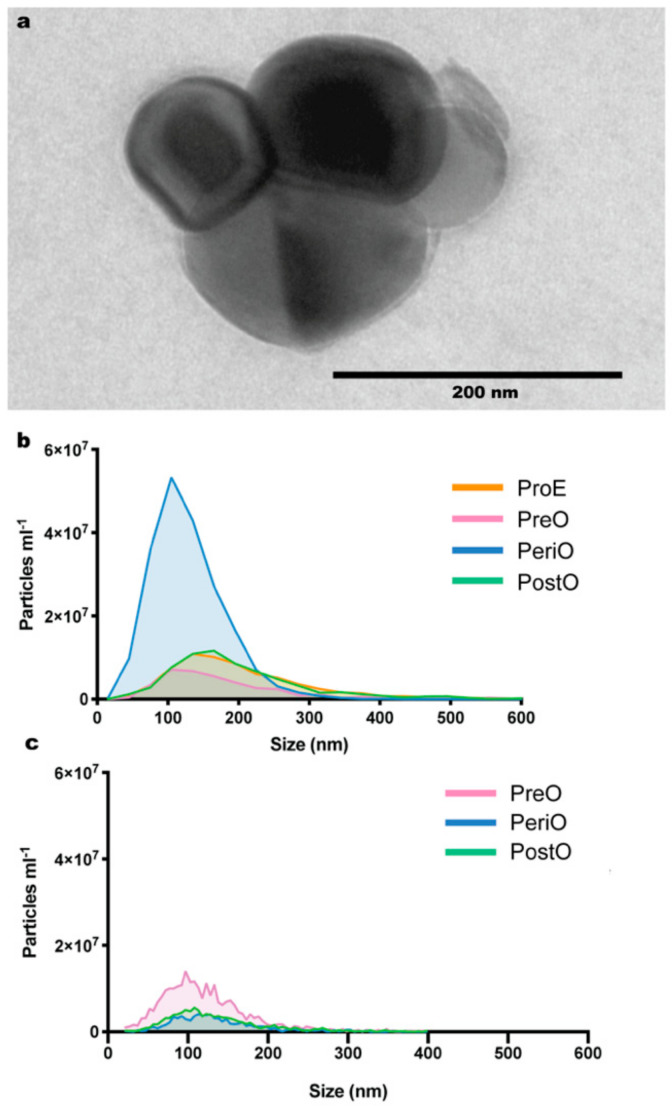
Cat and dog oviductal extracellular vesicle (oEV) analysis. In (**a**), transmission electron microscopy of dog oEVs, showing the characteristic doughnut shape. oEV concentration and size distribution analyzed by nanoparticle tracking analysis in different stages of the estrus cycle in dogs (**b**) and cats (**c**). ProE = proestrus; PreO = pre-ovulatory; PeriO = peri-ovulatory; PostO = post-ovulatory.

Here, we used the Total Exosome Isolation Kit (Invitrogen, Burlington, ONT, Canada) to recover dog and cat oEVs. This precipitation method was already shown to be superior to ultracentrifugation protocols in terms of EV isolation yield, without changes in EV quality [36]. Similar to our previous work in the cat [31], this commercially available kit was a reliable tool to recover dog oEVs. We used transmission electron microscopy (TEM) and nanoparticle tracking analysis (NTA) to confirm the presence, characterize and quantify the oEVs in our isolates. Specifically, TEM confirmed the presence of circular vesicles with the characteristic doughnut shape, in a heterogeneous sized population (Figure 1a). ZetaView (Particle Metrix Inc, Meerbusch, Germany) NTA showed the presence of EVs with an average size of 158.90 ± 33.73 nm and 140.5.3 ± 31.54, for dogs and cats, respectively (Figure 1b,c, respectively). The total oEV concentration detected by NTA ranged from 1.8 × 10^10^ to 1.8 × 10^11^ particles·mL^−1^, with an average of 4.5 ± 4.8 × 10^10^ particles·mL^−1^ in the dog, and from 2.0 × 10^10^ to 4.5 × 10^10^ particles·mL^−1^, with an average of 3.1 ± 1.3 × 10^10^ particles·mL^−1^ in the cat. There were no differences in oEV concentration (as determined by NTA) among reproductive stages, except for oEVs collected during the peri-ovulatory period (PeriO) having higher concentration of oEVs compared to other groups (Figure 1b). 

Ultraperformance liquid chromatography and tandem mass spectrometry (UPLC–MS/MS) were used to identify the protein content of dog and cat oEVs among different reproductive stages (proestrus, pre-ovulatory, peri-ovulatory, and post-ovulatory stages; see classification criteria in Materials and Methods). Across all reproductive stages, gene ontology (GO) analysis identified cellular component pathways that are related to EVs, including extracellular vesicle (GO:1903561), extracellular exosome (GO:0070062), and secretory vesicle (GO:0099503) in both dogs and cats (Supplementary Data 1). In particular, the dog and cat oEVs contained several EVs markers, such as cytosolic proteins (EHD1, EHD3, ANXA1, ANXA3, ANXA4, ANXA5, ANXA6, ANXA7, ANXA11, HSP90AB1, HSP90B, HSPA9, HSPA2, HSPA4, ACTA1, ACTA2, ACTB, ACTC1, TUBA4A, TUBB, and TUBB1), and transmembrane or lipid-bound extracellular proteins (CD9, CD63, CD5LGNAI1, GNAI3, GNAL, GNB1, GNB2, GNB4, and LAMP1), confirming the EV origin of the recovered oviductal samples [37]. 

### 2.2. oEV Protein Content Varies between Dogs and Cats

**Figure 2:**
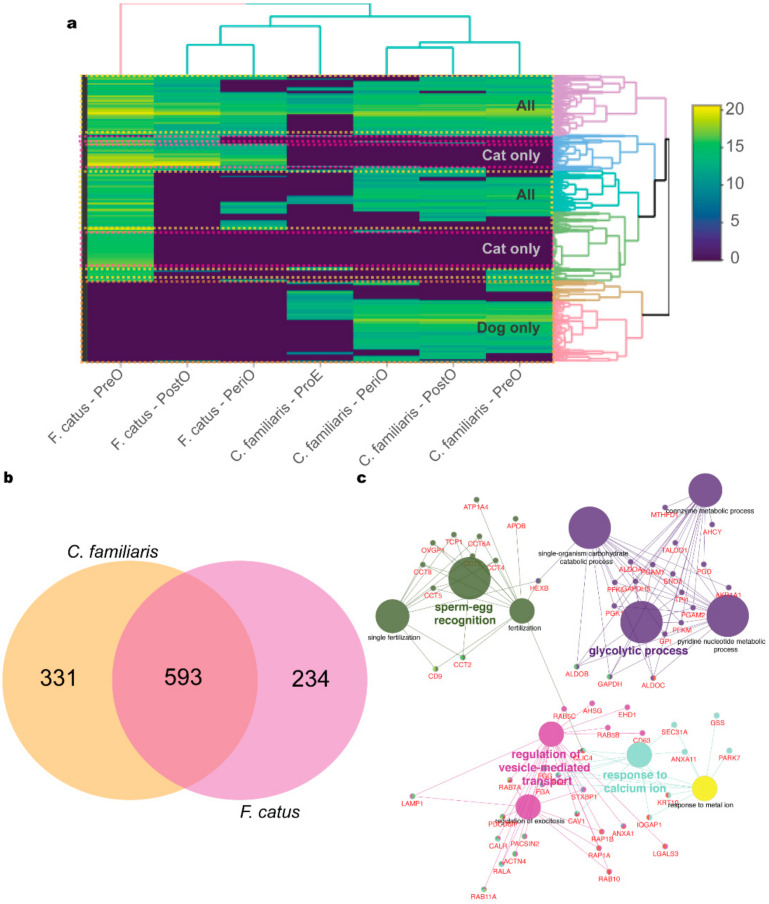
Profile of dog (*Canis familiaris*) and cat (*Felis catus*) oEV protein content. In (**a**), heatmap of oEVs at the different stages of the reproductive cycle: proestrus (ProE); pre-ovulatory (PreO); peri-ovulatory (PeriO); post-ovulatory (PostO). Note the division of proteins present in both dogs and cats (yellow, all), dog only (orange) and cat only (pink). In (**b**), Venn diagram of proteins present in dog and cat oEVs. In (**c**), GO terms for biological processes related to fertilization and sperm function modulated by proteins present in both dog and cat oEVs (all).

**Figure 3:**
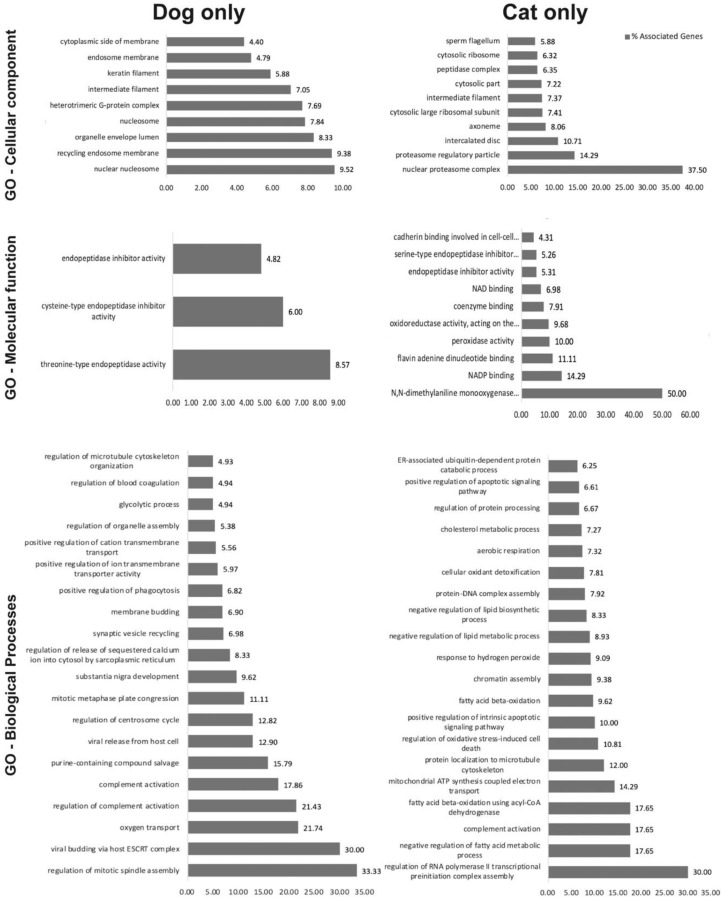
Top enriched gene ontology (GO) of cellular components, molecular function, and biological process terms for unique proteins in the dog and cat oEVs, analyzed by the Cytoscape plug-in ClueGO (*p* < 0.05) [38].

We first separated the 1158 identified proteins into three groups, independent of their reproductive stage: (1) proteins shared by dog and cat oEVs (all: 593 proteins); (2) proteins identified only in cat oEVs (cat only: 234 proteins); (3) proteins present only in dog oEVs (dog only: 331 proteins), as depicted in Figure 2a,b (Supplementary Data 2). The species-specific proteins were further processed for GO analysis using the Cytoscape plug-in ClueGO [38]. From the proteins common to both dogs and cats (All), GO for biological process pathways related to fertilization and sperm function were identified (Figure 2c). Analysis of the GO for cellular components revealed 9 terms for dogs and 11 for cats (Figure 3, Supplementary Data 3). Regarding GO for molecular function, three terms were identified for dogs—all of which were endopeptidases—and 12 terms related to cell adhesion were found in the cat (Figure 3, Supplementary Data 3). Our GO analysis also identified 26 terms specific to the dog and 36 to the cat (Figure 3, Supplementary Data 3). Next, we used the GO pathways (biological processes and molecular function) to categorize oEV proteins that could prevent/overcome sperm cryoinjuries. Proteins from all, dog only, and cat only were then assigned to six groups based on their targets or protective mechanisms: plasma membrane (120 proteins), mitochondria (205 proteins), acrosome (115 proteins), stress response (50 proteins), motility (97 proteins) and DNA/RNA (274 proteins; Supplementary Data 4). 

### 2.3. oEVs Improve Sperm Post-Thaw Motility in the Red Wolf but Not in the Cheetah 

**Figure 4:**
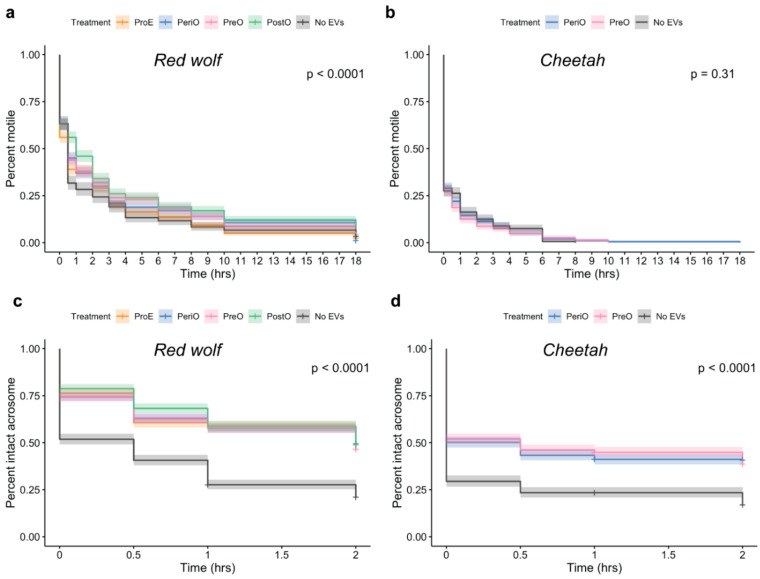
Total motility and acrosome integrity of red wolf and cheetah sperm thawed in the presence or absence (no EVs) of dog and cat oEVs, respectively, collected at different reproductive stages. In (**a**), post-thaw motility data of red wolf and in (**b**) of cheetah sperm analyzed at 0, 0.5, 1, 2, 3, 4, 6, 8, 10 and 18 h of incubation. Acrosome integrity of red wolf (**c**) and cheetah (**d**) sperm was evaluated at 0, 0.5, 1 and 2 h after thawing. All data are presented as point estimate ± 95% confidence interval. ProE = proestrus; PreO = pre-ovulatory; PeriO = peri-ovulatory; PostO = post-ovulatory.

Red wolf and cheetah sperm were thawed in the presence or absence of oEVs recovered from dogs or cats, respectively, during the proestrus (ProE), pre-ovulatory (PreO), peri-ovulatory (PeriO) or post-ovulatory (PostO) stages. The overall total motile cryopreserved red wolf sperm decreased from 80 ± 10% (fresh) to 61 ± 18% (immediately post-thaw; *p* = 0.0782, t-test). Post-thaw total motile red wolf sperm incubated in dog PeriO or PostO oEVs was significantly higher than the control (no oEVs) (*p* = 0.0464 for PeriO vs. no oEVs and 0.0003 for PostO vs. no oEVs, chi-square test; Figure 4a). Overall, the total motility of cryopreserved cheetah sperm significantly decreased from 65 ± 12% (freshly collected) to 27 ± 6% (immediately post-thaw, *p* = 0.0002, *t*-test). The presence of cat oEVs during thawing did not influence the post-thaw motility of cheetah spermatozoa, which decreased to less than 10% after 4 h incubation in all treatment groups (Figure 4b). 

### 2.4. oEVs Prevents Premature Acrosome Reaction of Red Wolf and Cheetah Spermatozoa

Red wolf sperm thawed in the presence of dog oEVs displayed significantly higher proportions of sperm with intact acrosomes compared to the control (no oEVs) (*p* < 0.0001 for all stages vs. no oEVs, chi-square test; Figure 4c). Furthermore, there were no differences in the percentages of intact acrosomes among samples exposed to oEVs of varying reproductive stages (*p* = 0.8069 for ProE vs. PreO, 0.2825 for ProE vs. PeriO, 0.1838 for ProE vs. PostO, 0.1915 for PreO vs. PeriO, 0.1266 for PreO vs. PostO and 0.7628 for PeriO vs. PostO, chi-square test). The presence of cat oEVs during thawing also better supported the post-thaw acrosome integrity of cheetah spermatozoa compared to the no oEV control (*p* < 0.0001 for all stages vs. no-oEVs, chi-square test; Figure 4d). Similarly, reproductive stages did not influence the ability of oEVs to sustain the integrity of the acrosomal membranes in cheetah sperm (*p* = 0.8883 for PostO vs. PreO).

## 3. Discussion

Extracellular vesicles (EVs) are membrane-encapsulated particles containing regulatory molecules that contribute to cell–cell communication by carrying proteins, peptides, RNA species (microRNAs, mRNAs, and long non-coding RNAs), lipids, and DNA fragments [39,40,41]. EVs have been isolated from prostate, epididymal, uterine, follicular, and oviductal fluids, and shown to support or promote function of targeted cells [32,33,42,43,44,45,46,47,48,49]. EVs secreted by the oviduct (oEVs) facilitate gamete function, fertilization and embryo development [31,32,44,45,46,50]. Specifically, studies have shown that oEVs improve gamete maturation and/or embryo production in vitro in the cat, mouse, cow and human [31,32,33,34,45,51,52]. Furthermore, the presence of exosomes secreted by adipose-derived mesenchymal stem cell in freezing media improved post-thaw motility as well as the integrity of the plasma and acrosomal membranes of dog and rat sperm [53,54]. However, when considering the use of banked samples for genetic rescue of endangered species, there is still a need to identify methods of improving post-thaw function of sperm that have been stored for many years or even decades. Recently, our group has shown that oEVs bind to the acrosome and mid-piece of cat spermatozoa which resulted in improved sperm motility, maintained acrosome integrity and increased sperm fertilizing ability [31]. Here, we showed that dog and cat oEVs carried proteins that could potentially support sperm function. Further, we demonstrated that thawing sperm in the presence of oEVs improved sperm motility in the red wolf, and sustained acrosome integrity in both red wolf and cheetah sperm.

The most effective way of preserving mammalian sperm long term is through cryopreservation [17,55,56,57]. However, freezing and thawing procedures are known to cause damages that impair sperm function and survival in several species [25,27,55,58,59,60,61,62]. Sperm cryoinjuries can include insults to the plasma membrane (integrity, changes in ion channels and lipid compositions, and alterations of membrane fluidity), nucleus (DNA fragmentation), perinuclear theca (changes to cytoskeletal proteins and nuclear decondensation), mitochondrial function (reduced activity, increased lipid peroxidation), acrosome (premature exocytosis), motility (reduction), mRNAs and microRNAs (degradation), and tyrosine phosphorylation of sperm proteins [27,28,60,63,64,65,66,67]. Previous studies have demonstrated that cheetah sperm is highly sensitive to the thawing and cryoprotectant removal processes, which cause a more than 35% reduction in sperm with intact acrosomes and more than a 15% decline in gamete motility [25,28]. Similarly, cryopreserved red wolf spermatozoa exhibit 50% and 40% decline in motility and acrosome integrity, respectively, compared to fresh ejaculates [27]. In the present study, we observed a 19 ± 12% and 37 ± 9% decrease in motility immediately post-thaw for red wolf and cheetah sperm, respectively. However, the use of dog oEVs during thawing sustained red wolf motility during the 18 h incubation compared to the no oEV control. This finding is consistent with a previous study where incubating dog sperm with EVs obtained from adipose-derived mesenchymal stem cells improved post-thaw motility [53]. The impact of oEVs on post-thaw motility appeared to be species-specific. Specifically, we did not observe the beneficial effect of cat oEVs on post-thaw motility in the cheetah. This was likely due to differences in the susceptibility to cryoinjuries between the cheetah and red wolf rather than a deviation in the function of dog and cat oEVs, as thawing of cheetah sperm in the presence of dog oEVs (PostO) displayed no improvements on cheetah sperm motility (data not shown, *n* = 3 males). Because the species’ low heterozygosity [5], male cheetah consistently ejaculate sperm with high proportions of morphologically abnormal gametes (>60%) [68,69], whereas red wolf ejaculates contain 50% to 60% normal cells [27]. Previous studies have shown that sperm from teratospermic donors (>60% of cells in the ejaculates are abnormal) are more susceptible to cryoinjury than normospermic counterparts [70]. 

A vital event for fertilization in mammals is the ability of spermatozoa to undergo acrosomal exocytosis (AE) [71]. AE occurs during the sperm transit through the oviduct, and is modulated by the oviductal and follicular fluids, cumulus oophorous cells, and the zona pellucida [20,71,72]. During freezing and thawing, the membranes of spermatozoa undergo structural alterations, such as swelling or vesiculation, that resemble the process of the physiological acrosome reaction, as revealed by transmission electron microscopy [73]. This premature acrosome reaction is undesirable, since acrosome reacted spermatozoa are not able to bind and fertilize the oocyte [74]. Here, we demonstrated that both red wolf and cheetah sperm thawed in the presence of oEVs had higher proportions of intact acrosomes than the non-supplemented control. This finding is consistent to that observed in dog and rat sperm cryopreserved in the presence of mesenchymal stem cells EVs [53,54]. In the cow, supplementation of oEV to post-thaw sperm promoted calcium ionophore-induced AE [35]. Together, these results indicate that EVs from both reproductive and non-reproductive sources contains key factors that protect sperm against cryoinjuries or restores cell function after thawing. 

Interestingly, only PeriO and PostO dog oEVs supported red wolf sperm motility, while the positive effect on acrosome integrity was seen in all oEV groups. Unlike other domestic mammals, which ovulate metaphase-II stage oocytes, canine ovulation is characterized by the release of an immature oocyte that requires an additional 3–4 days to undergo maturation in the oviduct prior to fertilization [75]. Furthermore, studies have shown that domestic dog sperm maintain motility and fertilizing ability for up to 9–11 days in the female reproductive tract [76]. Little is known about oocyte maturation and fertilization in wolves, but what is known in the domestic dogs likely can be applied to the wild counterparts. Considering that sperm must be motile to fertilize the oocyte during the peri- and post-ovulation periods [77,78], it is possible that PeriO and PostO dog oEVs carry key factors (such as RNAs, lipids and proteins) that support sperm motility as observed in the present study. 

In the present study, we found that 51 proteins were exclusively expressed in PeriO and PostO dog oEVs (for all differentially expressed proteins in cat and dog oEVs from the different reproductive stages see Supplementary Data 5). Among these unique proteins, the Testis-specific gene antigen10 (Tsga10) was the most abundant protein. An increase in the *Tsga10* gene expression was shown to improve sperm morphology and motility in mice [79]. Another identified protein was the Cysteine-Rich Secretory protein-3 (Crisp-3), which was already associated with higher motility [80], and as a possible marker of stallion sperm freezeability [81]. Crisp-2 was also unique to these stages, and it was exclusively detected in the dog oEVs. It has been shown that Crisp-2 is present in the sperm acrosome and tail, and Crisp-2-deficient mouse sperm possess a stiff midpiece; as a result, these sperm are not able to exhibit rapid progressive motility [82]. Furthermore, Crisp-2 can bind to the Catsper ion channel, which is necessary for normal sperm motility [82]. Another unique protein identified was the Plasma Membrane Ca^+2^ ATPase 4 (ATP2B4 or PMCA4), PMCA4, a member of a family of calcium efflux pumps. PMCA4 plays crucial roles in calcium homeostasis in mouse sperm by maintaining low resting cytosolic calcium and a calcium gradient across the sperm plasma membrane [3]. PMCA4-knock out mice have severely impaired sperm motility [83], and it was already demonstrated that oEVs can deliver PMCA proteins to mouse spermatozoa [33]. As such, it is evident that multiple proteins with known roles in promoting sperm motility are present in these stage-specific dog oEVs. 

Dog and cat oEVs obtained from all reproductive stages sustained acrosomal integrity of red wolf and cheetah sperm, respectively, during in vitro incubation. Therefore, we postulate that proteins present in oEVs of all reproductive stages in both species could mediate this protective effect. A total of 87 proteins were identified in both dog and cat oEVs of all estrus stages (Supplementary Data 5). Among them is the heat shock protein A8 (HSPA8), which is a highly conserved member of the Hsp70 family. HSPA8 is expressed in oviductal cells, is secreted into oviductal fluid in the cow [84,85], and has previously been identified in the dog post-ovulatory oviductal proteome [86]. It has been shown that HSPA8 attaches to the sperm surface during sperm transport in the cow, and incubating sperm with this protein improves sperm motility and membrane integrity in the bull, boar, brown bear and ram [84,87,88,89,90]. In particular, HSPA8 maintains plasma membrane integrity and prevents premature AE in bull and brown bear, when added to cryopreservation medium [84,90]. Another protein common to all oEVs is the Actin-Related Protein 2/3 Complex Subunit 4 (Arpc4), which is a component of the Arp2/3 complex. Arp2/3complex is critical for the regulation of actin polymerization, which is associated with sperm motility and capacitation status [91]. Inhibition of the Arp2/3 complex in the mouse sperm using the CK-636 inhibitor induced AE and decreased cleavage and blastocyst formation in a dose-dependent manner [91]. Although not investigated in the present study, the delivery of one or more of these proteins to post-thaw red wolf and cheetah sperm could explain the observed maintenance in acrosome integrity and motility during in vitro incubation.

In addition to proteins, oEVs also contain lipids and RNAs that can contribute to the protective effects observed in this study. The delivery of oEVs lipids can have a protective role on sperm thawing by modulating their plasma membrane properties, such as permeability and fluidity. EVs originating from different cell types (PC-3 cells, Oli-neu cells, HepG2/C3a cells, B-Lymphocytes, mast cells, dendritic cells, reticulocytes, platelets, and adipocytes) have a bilayer membrane rich in cholesterol, sphingomyelin, glycosphingolipids, and phosphatidylserine [92]. To date, there is little information on lipid composition of EVs in reproductive biofluids. Most studies have been focused on the analysis of seminal plasma-derived EVs in the human, horse, and pig [93,94,95]. It has been shown that the lipid composition of reproductive tract EVs are very similar to other cell secreted EVs [92], which lead us to postulate that female reproductive biofluid derived-EVs would also be enriched in cholesterol, sphingomyelin, glycosphingolipids, and phosphatidylserine. An increased delivery of cholesterol from the oEVs to the sperm could stabilize the sperm membrane, preventing membrane damage and premature AE during thawing. Increased membrane cholesterol has been described to have a stabilizing effect, preventing sperm membrane damage and premature AE in different species, including pig, goat, bull, dog and horse [84,96,97,98]. Collectively, the modulation of sperm membrane lipids by EVs during cryopreservation represents an intriguing area for further exploration in this field. 

We demonstrated that dog and cat oEVs carry proteins important for sperm function, including proteins that have the potential to restore sperm function after cryopreservation. Thawing red wolf and cheetah sperm in the presence of dog and cat oEVs, respectively, improved post-thaw motility in the red wolf and prevented premature acrosome exocytosis in both red wolf and cheetah sperm. Future studies should investigate whether these positive effects can be maximized by incubating sperm with oEVs during the cryopreservation process, and whether they can improve fertilizing ability of frozen-thawed gametes. Nevertheless, the use of oEVs can be a valuable tool to improve sperm cryosurvival and, consequently, the success of artificial insemination with frozen-thawed samples in these threatened carnivores, representing a step forward for successful assisted breeding of these species. 

## 4. Materials and Methods 

### 4.1. Animal Handling and Semen Collection 

All animal procedures were approved by the Institutional Animal Care and Use Committee of the Smithsonian’s National Zoological Park (IACUC numbers: red wolf: #18-05 and cheetah: #09-41, #15-30 and #16-26). Adult, male red wolves (*n* = 5) were maintained at three institutions in the USA and were sampled during the breeding season (February and March 2018 and 2019). The age of sperm donors ranged from 2 to 9 years old at the time of semen collection. Animals were housed singly, in breeding/companion pairs, with multiple male conspecifics, or in family units (packs). All institutions adhered to the husbandry guidelines put forth by the Red Wolf Species Survival Plan^®^ (SSP), including housing animals in enclosures containing natural substrate, foliage, and sheltered dens with bedding material. Adult male cheetahs (*n* = 5) were maintained at one institution in the USA and were sampled between 2011 and 2019. The age of sperm donors utilized in this study ranged from 3 to 12 years old at the time of semen collection. Animals were housed singly or a group of brothers (coalition). Cheetahs received a commercially produced beef-based diet 5 days per week and supplements of whole rabbits and bones 2–3 days per week. The institution adhered to the husbandry guidelines put forth by the Cheetah Species Survival Plan^®^ (SSP), including housing animals in pens of approx. 18,000 ft^2^ containing natural substrate, foliage, and sheltered dens with bedding material. 

Adult red wolves were anesthetized by veterinary staff at participating institutions. The choice of anesthetic drug was under the discretion of each attending veterinarian, and the majority of procedures were carried out using butorphanol (0.4 mg/kg im) with medetomidine (0.04 mg/kg im) or dexmedetomidine (0.02 mg/kg im), sometimes also including midazolam and maintained with isoflurane as needed. Prior to the start of electroejaculation, the rectum was evacuated using a well-lubricated gloved hand and the penis carefully exteriorized and washed thoroughly with sterile saline and wiped dry. To avoid urine contamination in semen samples, the urinary bladder was emptied by inserting a 3.5 fr × 22 in polypropylene urinary catheter into the urethra and urine aspirated and bladder flushed with saline solution. Semen collection was performed using an electroejaculation method as previously described for the red wolf [27] via insertion of a lubricated rectal probe (1.9–2.1 cm) and application of about 60 to 90 total electrical stimulations (2–6 volts) over a period of 20 min. Intensity of the stimulation was adjusted as appropriate based on individual animal’s physical response. Ejaculates were collected into sterile polypropylene specimen cups and were assessed for concentration (using a hemocytometer), % motile sperm (total motility), volume, morphology and processed by cryopreservation as described below.

Adult male cheetah fasted for approximately 24 h before inducing anesthesia by the institution veterinary staff using an intramuscular injection of a combination of the following drugs (as deemed most appropriate by the attending veterinarian): medetomidine hydrochloride (22.0–25.0 μg/kg body weight), midazolam (0.2 mg/kg body weight), and/or ketamine HCl (2.0–3.5 mg/kg). Propofol (0.5–4.0 mg/kg, iv) was administered, as necessary, to maintain an appropriate plane of anesthesia during electroejaculation. Each ejaculate collection and subsequent evaluation were performed according to previously described, rigorous protocols and metrics [25,99]. Briefly, this involved using a standardized set of three stimulation series with a total of 80 stimulations over an approximate 30 min interval to collect cheetah semen into sterile vials which were assessed for concentration (using a hemocytometer), total motility, volume, morphology and processed by cryopreservation as described below. 

### 4.2. Sperm Cryopreservation

After assessing for initial total motility, red wolf sperm concentration was determined using a hemocytometer. Ejaculates were centrifuged at 300× *g* for 5 min and supernatant removed and samples processed for cryopreservation as previously described [27]. Following centrifugation, sperm were resuspended in TRIS-egg yolk extender without cryoprotectant to a concentration of 200 × 10^6^ sperm·mL^−1^. The samples were chilled at 5 °C for at least 30 min. Then, an equal volume of extender containing 8% glycerol (*v*/*v*) was added dropwise over 2–3 min to the chilled samples, for a final cryoprotectant concentration of 4% glycerol, and final sperm concentration of 100 × 10^6^ cell·mL^−1^. Chilled samples were then frozen using a dry ice pelleting method [27]. Briefly, aliquots (~20–30 µL) of the chilled semen were pipetted using pre-cooled pipettes into indentations in a block of dry ice. After 3–4 min, the pellets were plunged into liquid nitrogen, after which they were loaded into labeled cryovials and stored in liquid nitrogen until use.

After assessing for initial motility and forward status, cheetah sperm concentration was determined using a hemocytometer. Samples were centrifuged for 8 min at 100× *g* to concentrate the sperm pellet and remove the seminal fluid. After centrifugation, the pellet was resuspended in Test Yolk Buffer (TYB; Irvine Scientific, Santa Ana, CA, USA) with 0% glycerol in a sterile Eppendorf tube. Final sperm concentration was either 50 or 60 × 10^6^ total motile sperm·mL^−1^. Samples were placed into a water bath and cooled to 5 °C (approx. 2.5 h) in a walk-in refrigerator. After reaching 5 °C, an equal volume of TYB with 8% glycerol was added to the sample step-wise over a period of 30 min (add ¼ volume and wait 15 min, add ¼ volume and wait 15 min, and add the last ½ volume). Samples were loaded into 0.25 cc sterile straws (~120 µL/straw) and cryopreserved over liquid nitrogen vapor. Straws were placed 7.62 cm above the liquid for 1 min, then 2.54 cm above the liquid for 1 min and then plunged into the liquid nitrogen. 

### 4.3. Oviductal EV (oEV) Isolation

Post-pubertal domestic dog and cat oviducts (*n* = 11 and 9, respectively; 11 months to 4 years old) were opportunistically collected from local veterinary clinics after routine ovariohysterectomy of stray and household cats and dogs and transported at 4 °C to the laboratory within 6 h of excision. For the domestic dogs, all were designated as “in heat” by veterinary staff, based on appearance of the vulva (swollen and/or serosanguinous discharge). As previously described [31], after being washed three times in Phosphate Buffer Saline solution (PBS, GIBCO, USA), a 23G needle was inserted through the fimbria opening and the entire oviduct was flushed with 2 mL and 1 mL of PBS, for dog and cat oviducts, respectively. The flush was centrifuged at 2000× *g* (room temperature) for 30 min to remove cells and debris, then the supernatant was mixed with 1000 (dog) and 500 (cat) μL of the Total Exosome Isolation Reagent (Invitrogen, Burlington, ONT, Canada) and incubated overnight at 4 °C. The samples were centrifuged at 10,000× *g* for 1 h, and the pellet resuspended in 50 μL of PBS. EVs were then aliquoted and kept at −20 °C until use.

oEV samples were categorized into different reproductive stages, which were determined based on ovarian morphology: (1) proestrus (ProE)—absence of corpus luteum and presence of follicles smaller than 2 mm of diameter; (2) pre-ovulatory (PreO)—absence of corpus luteum and presence of follicles bigger than 3 mm of diameter; (3) peri-ovulatory (PeriO)—corresponded to the period immediately after ovulation, determined by the presence of *corpora hemorrhagica* with ovulatory foci; (4) post-ovulatory (PostO)—determined by the presence of one or more corpora lutea without being possible to identify the ovulatory foci [100,101,102]. 

### 4.4. oEVs Quantitation

Nanoparticle tracking analysis was performed using the ZetaView S/N 17–332 (Particle Metrix, Meerbusch, Germany) and data were analyzed using this software (ZetaView 8.04.02, Particle Metrix, Meerbusch, Germany) by Alpha Nano Tech (Morrisville, NC, USA) as previously described [36]. Each oEV sample (n  =  11 and 9 dog and cats, respectively) was diluted 100× in PBS and loaded into the cell, and the instrument measured each sample at 11 different positions throughout the cell, with three cycles of readings at each position. The pre-acquisition parameters were: sensitivity of 85, frame rate of 30 frames per second (fps), shutter speed and laser pulse duration of 100, temperature of 19.81 °C, and pH of 7.0. Post-acquisition parameters were set to a minimum brightness of 22, a maximum area of 1000 pixels, and a minimum area of 10 pixels [36]. All parameters (temperature, conductivity, electrical field, and drift measurements) were documented for quality control. After software analysis, the mean, median, and mode (indicated as diameter) sizes, as well as the concentration of the sample, were calculated, excluding outliers [36]. The number of particles per particle size curves was created using quadratic interpolation [36].

### 4.5. oEVs Proteomic Analyses

Oviductal EVs for each estrous stage (n  =  three animals for each stage, except dog immediately post-ovulation which had two animals) were pooled to a final concentration of 2 × 10^10^ particles·mL^−1^, and frozen at −20 °C. Proteins were extracted and prepared via single-pot, solid phase-enhanced sample-preparation (SP3) technology [103], and analyzed using ultraperformance liquid chromatography and tandem mass spectrometry (UPLC—Thermo Easy-nLC 1200 fitted with a heated, 25 cm Easy-Spray column—MS/MS—Thermo Q-Exactive HF-X quadrupole-Orbitrap mass spectrometer, ThermoFisher Scientific, San Jose, CA, USA) by Bioproximity LLC (Chantilly, VA, USA). The peptide datasets (mzML format) were exported to Mascot generic format (.mgf) and searched using X!!Tandem (https://www.thegpm.org/TANDEM) [104] using both the native and k-score scoring algorithms [105], and by OMSSA [106]. RAW data files were compared with the protein sequence libraries available for the domestic cat (*Felis catus*, taxa 9685) and dog (*Canis familiaris*, taxa 9615). Label-free quantification (MS1-based) was used, and peptide peak areas were calculated using OpenMS (https://www.openms.de/) [107]. Proteins were required to have one or more unique peptides across the analyzed samples, with *E*-value scores of 0.01 or less.

### 4.6. Functional GO Clustering

Data Entrez Gene IDs were mapped for all identified proteins using the R package rentrez (ver 1.2.1) [108]. The background dataset for all analyses were the dog (*Canis familiaris*) and the cat (*Felis catus*) genomes. Because protein expression levels were determined via UPLC–MS/MS, small differences in sample concentrations and/or measurement error can introduce artefactual differences across samples. To control for this effect, we used Probabilistic Quotient Normalization (PQN) [109], which has been shown to have the best performance of all routinely used normalization methods [110]. After PQN transformation, we filtered out proteins with fewer than 100 counts per million (1018 proteins). We then utilized R package ‘NOISeq’ (v 2.30.0) [111] to identify whether there was any differential expression of proteins between species and/or reproductive stages.

The Cytoscape 3.5.1 plugin ClueGO (v.2.5.6) [38] was used to visualize interactions of EV proteins and networks integration by gene ontology (GO) terms “biological processes”, “molecular function” and “cellular components” using the *Canis familiaris* and the *Felis catus* genomes. The evidence was set to “Inferred by Curator (IC)”, and the statistical test was set to a right-sided hypergeometrical test with a κ score of 0.7–0.9 using Bonferroni (step down). The function “GO Term fusion” was selected, the GO term restriction levels were set to 3, and a minimum of 3 genes or 5% genes in each GO term were used.

### 4.7. oEV Transmission Electron Microscopy

TEM preparation and imaging was performed at the Alpha Nano Tech (Morrisville, NC, USA). Briefly, oEVs (10 μL) were dropped onto Formvar-coated carbon-coated TEM grids (Electron Microscopy Sciences, Hatfield, PA, USA), allowed to be adsorbed to the grid for 10 min, and washed two times with water (30 s each). A 2% Aqueous Uranyl Acetate solution was applied to the grid for 30 seconds (negative staining of exosomes), then whisked off with filter paper, and grids were air dried before imaging on a Jeol JEM1230 transmission electron microscope (Jeol USA Inc., Peabody, MA, USA).

### 4.8. Sperm Thawing

**Figure 5:**
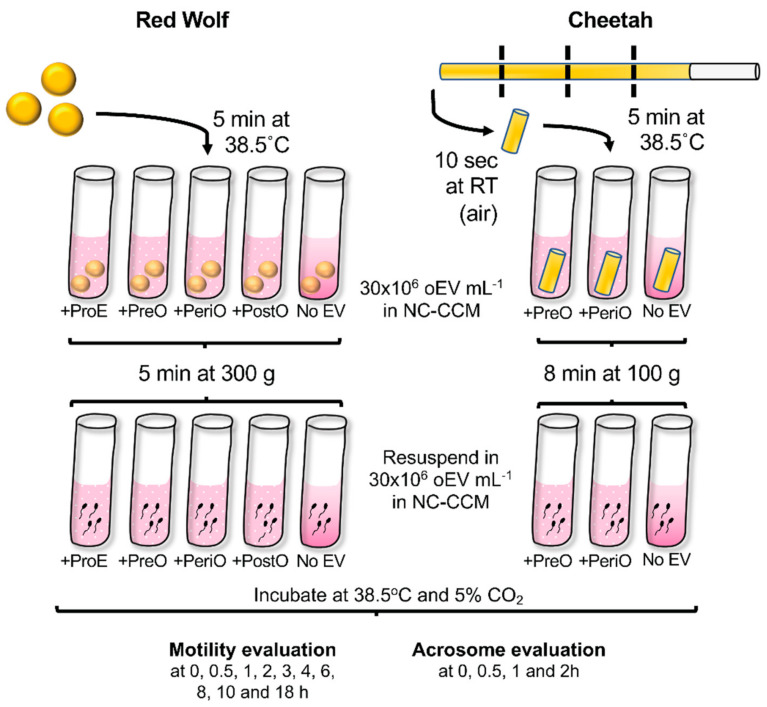
Experimental design of red wolf and cheetah sperm thawing with/without oEVs. NC-CCM = non-capacitating canine capacitation medium; ProE = proestrus; PreO = pre-ovulatory; PeriO = peri-ovulatory; PostO = post-ovulatory.

Red wolf cryopreserved sperm pellet was thawed in 1 mL of non-capacitating canine capacitation medium (NC-CCM: 106.33 mM NaCl, 25 mM HEPES, 0.5 mg·ml^−1^ bovine serum albumin, 4.78 mM KCL, 1.71 mM CaCl_2_, 1 mM MgCl_2_, 1.19 mM KH_2_PO_4_, 0.25 mM Na pyruvate, 21.55 mM Na lactate, 2.78 mM glucose, 0.05 g L^−1^ *Streptomycin* sulphate and 100,000 units of *Penicillin G;* modified from [112]) at 38.5 °C, with (30 × 10^6^ particles·mL^−1^ dog oEVs collected from each of the four reproductive stages—ProE, PreO, PeriO and PostO) or without oEVs for 5 min. Sperm were then centrifuged at 300× *g* for 5 min. The sperm pellet was resuspended in NC-CCM either without dog oEVs or supplemented with 30 × 10^6^ particles·mL^−1^ dog oEVs (ProE, PreO, PeriO and PostO), to achieve a final sperm concentration of 5 × 10^6^ sperm·mL^-1^ dog sperm. All treatment groups were kept in an incubator at 38.5 °C, 5% CO_2_ in air, and then analyzed for total sperm motility and acrosome integrity. Cheetah sperm straws were cut in four pieces (while submerged in liquid nitrogen), and each piece was thawed for 10 sec in air and then in 1 mL of NC-CCM at 38.5 °C, with (30 × 10^6^ particles·mL^−1^, for each of oEVs collected from one of two reproductive stages—PreO and PeriO) or without cat oEVs. Sperm were then centrifuged at 100× *g* for 8 min and the pellet resuspended in NC-CCM at 38.5 °C, with (30 × 10^6^ particles·mL^−1^, of respective oEVs) or without cat oEVs to a final sperm concentration of 5 × 10^6^ sperm·mL^−1^, and kept in an incubator at 38.5 °C, 5% CO_2_ in air and analyzed for total sperm motility and acrosome integrity (Figure 5). 

### 4.9. Sperm Motility and Acrosomal Analyses

Sperm samples were incubated at 38.5 °C in thawing medium with or without oEVs for up to 24 h and samples were collected at 0, 0.5, 1, 2, 3, 4, 6, 8, 10 and 18 h for motility analysis and at 0, 0.5, 1 and 2 h for acrosome integrity analysis. For motility analysis, 3 μL of sperm samples were dropped onto a glass slide, covered with a coverslip and observed at a magnification of 200× on a positive phase-contrast microscope (EVOS FL auto 2, Invitrogen, Bothell, WA, USA) with a warmed (38 °C) stage. At least five fields per sample were recorded, and a minimum of 200 spermatozoa were assessed for total motility (% of total sperm moving, independent of moving pattern) by two different researchers, who were unaware of the treatment (blinded evaluation). 

For acrosome integrity assessment, 5 μL of sperm were fixed in 5 μL of 4% paraformaldehyde for 15 min and labeled with HOECHST33342 (for DNA, 5 µg·mL^−1^; Thermo Fisher, Rockford, IL, USA) and the acrosome-specific fluorochrome fluorescein isothiocyanate-labeled peanut (*Arachis hypogaea*) agglutinin (FITC-PNA, 10 µg·mL^−1^; Thermo Fisher, Burlington, ONT, Canada). At least two hundred spermatozoa were counted per animal, treatment, and time point at 1000× magnification (EVOS FL auto 2, Invitrogen, Bothell, WA, USA).

To assess the effect of oEV treatment on sperm total motility, we fit non-parametric Kaplan–Meier survival functions [113] to the motility data, where the probability of maintaining motility to time *t, M_t_,* was calculated as:

(1) $$M_{t}=\underset{i:t_{i}\leq t}{\prod }\left(1-\frac{d_{i}}{n_{i}}\right)$$

where *n_i_* represents the number of sperm remaining motile until time *t_i_*, and *d_i_* the number of sperm that lost motility at time *t_i_*. Because we could not follow individual sperm across all six time points, we assumed a uniform distribution of motile vs. non-motile sperm throughout the sample, and defined a total sample size of 200 sperm (i.e., the minimum number counted at each time point). This allowed us to use the percentage of sperm remaining motile and the number of newly non-motile sperm at each time point as estimates of *n_i_* and *d_i_*, respectively. We then used log-rank test statistics to determine whether there was any significant effect of oEV treatment on sperm motility. Survival functions were fit using the R package ‘survival’ (v 3.1-8) [114].

### 4.10. Availability of Data and Materials

The datasets generated during the current study are available in the Figshare repository, under DOI: https://doi.org/10.6084/m9.figshare.12100755.v1.

## Supplementary Materials

The following are available online at https://www.mdpi.com/1422-0067/21/10/3733/s1, Supplementary Data 1–5.

## Abbreviations

AE	Acrosomal exocytosis
AI	Artificial insemination
ART	Assisted reproductive technologies
EVs	Extracellular vesicles
GO	Gene ontology
NC-CCM	Non-capacitation canine capacitation medium
NTA	Nanoparticle tracking analysis
oEVs	Oviductal extracellular vesicles
PeriO	Peri-ovulatory
PostO	Post-ovulatory
PreO	Pre-ovulatory
ProE	Proestrus
TEM	Transmission electron microscopy
TYB	Test yolk buffer
